# Persisting demand and supply gap for maternal and newborn care in eastern Uganda: a mixed-method cross-sectional study

**DOI:** 10.1186/s12978-017-0402-6

**Published:** 2017-10-24

**Authors:** Rornald Muhumuza Kananura, Suzanne Namusoke Kiwanuka, Elizabeth Ekirapa-Kiracho, Peter Waiswa

**Affiliations:** 1 Department of Health Policy Planning and Management, Makerere University School of Public Health, Kampala, Uganda; 2 Department of Social Policy, London School of Economics and Political Science, London, UK; 3 Makerere University Centre of Excellence for Maternal and Newborn Health Research, Kampala, Uganda; 4 Global Health Division, Department of Public Health Sciences, Karolinska Institutet, Stockholm, Sweden

**Keywords:** Maternal and newborn, Supply and demand side factors, Health facility readiness, Uganda, And sub-Saharan Africa

## Abstract

**Background:**

The slow progress in reducing maternal and newborn death in low and middle-income countries is attributed to both demand and supply-side factors. This study assessed the changes in maternal and newborn services in health facilities as well as demand for maternal and newborn health services in Eastern Uganda.

**Methods:**

The health assessment data were collected in August 2013 and September 2015 in the districts of Kamuli, Pallisa, and Kibuku. We purposively collected data on the availability of services from 40 health facilities that provided maternal and newborn services. In addition, we conducted 24 focus group discussions (FGDs) with women and men; and 18 key informant interviews (KIs) with health workers.

**Results:**

On the supply side, most health facilities persistently lacked lifesaving medicines such as misoprostol, IV Ampicillin, IV Gentamycin, IV Metronidazole, Magnesium Sulphate, Ergometrine, Corticosteroids, ferrous Sulphate, Folic Acid, Combined ferrous, Benzyl penicillin, and Diazepam (IM or IV). Basic newborn equipment such as stethoscope, fetal scope, working baby scale, newborn suction devices, newborn resuscitation device, and thermometer were persistently not available in most of the health facilities. Binders for Kangaroo Mother Care, blanket to wrap newborn, baby warmer or heat lamp were persistently not available in at least 80% of the health facilities. Other equipment for the management of labor and abortions such as Manual vacuum aspirator for abortion care, blank partographs and vacuum extractor were not available in most of the health facilities including referral facilities at baseline and follow-up. On the demand side, the qualitative interviews exposed long distances and inadequate transport to the health facilities, inadequate information, poverty, and poor services at the health facilities as major factors that impede women to utilize/access maternal and newborn services.

**Conclusion:**

There are distinct influences on both demand and supply side, which restrain both health care uptake and its quality. The frequent disparity between the health facility readiness to provide services and the women readiness to utilize them needs to be addressed as the country intensifies its efforts to reduce maternal and newborn deaths through boosting facility deliveries.

## Plain English summary

The poor services at health facilities and demand factors such as poor transport systems, inadequate information, lack of access to finances and community behaviors may be responsible for the sluggish progress in reducing maternal and newborn death in most of the low and middle-income countries. In this study, we assessed changes in health facility services from 40 health facilities using health facility assessment data that was collected in August 2013 and September 2015. We also conducted 24 focus group discussions with women who had delivered in the last 12 months and their partners. We also conducted key informant interviews with 18 health workers.

Overall, parenteral antibiotics drugs such as oxytocin, parenteral anticonvulsants for preeclampsia and eclampsia drugs such as magnesium sulfate, which are lifesaving medicines, were not available in most of the health facilities in both years. In addition, basic equipment such as manual vacuum extraction and neonatal resuscitation were persistently missing in most of the health facilities. Services most at risk mother and newborns were persistently missing in most of the health facilities. Most of the health facilities continuously lacked skilled health workers responsible for maternal and newborn services. The facilities also continuously experienced inadequate maternity space, which led to the discharge of newly delivered mothers before the recommended time.

On the demand side, poor transport systems, poor services at the health facilities, inadequate information and poverty were mentioned as the factors that stop rural women from utilizing/accessing maternal and newborn service.

Providing essential drugs/equipment, health workers in health facilities and addressing community challenges that stop some of the women from delivering at the health facilities are therefore crucial.

## Background

Globally, 800 maternal and 7700 newborn deaths occur each day and an additional 7300 women experience stillbirths [1–3]. The maternal and perinatal deaths are higher in the low-and-middle-income countries (LMICs) [4–8]. In Uganda, maternal mortality has reduced by 23% from 438/100,000 to 336/100,000 live births [9, 10]. On the other hand, the newborn mortality rate is estimated at 27/1000 per live births and the rate has remained stagnant for at least 15 years [9, 10]. Because of that, Uganda is ranked number five and number six among African countries with high newborn death and high maternal deaths, respectively [11]. The factors that contribute to the slow reduction in maternal and unchanged newborn death rates are from persistent known demand and supply side health system challenges that affect both service utilization/accessibility and quality of care [12].

Like other LMICs, the maternal and prenatal deaths in Uganda occur from complications that occur during pregnancy, at the time of and after delivery [1]. The majority of maternal deaths are due to direct obstetric causes, whereas the main causes of newborn deaths are preterm birth complications, low birth weight, intrapartum conditions and infections [1, 13, 14]. In addition, stillbirth result from untreated infections such as syphilis [1, 4]. These complications result from delays in accessing and receiving appropriate service, which is attributable to both demand and supply health system’s factors. The maternal and newborn complications can be prevented given a continuum of care that addresses both demand and supply health system factors. On the supply side, quality of care given at the health facility is crucial in averting the maternal and newborn deaths. For instance, delivery under proper monitoring of the skilled provider with essential equipment and drugs has been indicated as an effective intervention in improving the newborn’s and mother’s survival [15]. However, in resource-limited settings, most women hardly access the quality of care regardless of the places of delivery.

In Uganda, most rural health facilities frequently lack qualified staff, experience inadequate equipment, as well as essential drugs [14]. It is worth noting that skilled health workers, essential equipment, and drugs are all necessary components for the provision of quality maternal and newborn health services. Therefore, the absence of one element affects the effectiveness of the healthcare system. Too often, most of the rural health facilities hardly have all these elements needed for delivery of quality healthcare services. In fact, in Uganda like many other LMIC, health facilities frequently lack lifesaving commodities to be able to provide maternal and newborn quality care services [16]. The 2013 and 2014 national service availability and readiness assessment (SARA) reports highlighted inadequacies in the availability of lifesaving drugs and equipment in most of the health facilities including higher-level referral health facilities [17]. Because of this, pregnant and newly delivered women and their newborns hardly receive timely care to manage the morbidities and other conditions that are major causes of mortality among women and newborns [16]. This may explain why the newborn deaths in such communities are not significantly different between community and health facility deliveries [18–20].

On the demand side, the major factors that influence health service utilization operate at individual, household and community level [12]. The individual factors include the level of education, age, occupation, income, religion, culture, and knowledge about the services. The household factors are partner’s/family members’ influence and their knowledge on maternal and newborn health care. The community factors are a geographical location, distance to the health facilities and access to transport among others. In Uganda, at least 30% do not deliver under assisted skilled health worker and more than 40% do not attend the recommended number antenatal care and postnatal care services [9]. Besides, there are variations in health facility utilization/accessibility across the regions and within the communities. For instance, three in ten rural resident women do not deliver from health facilities compared to one in ten urban resident women [9].

The weakness in the supply side leads to poor services, which also reduces the demand for these services [21, 22], thus appealing for a continuum of care that addresses both demand and supply health system challenges [12, 14, 21, 23]. This study, therefore, seeks to understand the changes in health facilities services in responding to the demand for maternal and newborn services in rural communities of Uganda. The study also assesses maternal and newborn service’s utilization factors in rural communities of Uganda. This study is important for policymakers, health providers and implementing partners to understand the community and facility level bottlenecks. This will contribute to the design and replication of effective maternal and newborn health care interventions.

## Method

### Study area and design

This mixed-methods study used two periods’ cross-sectional health facility assessment surveys that were conducted in August 2013 and September 2015; and qualitative data that was conducted in September 2015 in the three districts of Kamuli, Pallisa, and Kibuku in Eastern Uganda. The health facility utilization in these districts is estimated at an average of 70% [9]. In total there are 47 health centers accredited to provide maternal and newborn services, of which 39 are health center III, 4 health center IV and 4 hospitals [24]. These health centers serve a population of 1,075,242 people [25].

### Sample size determination and selection

To assess the changes in facility services, we analyzed health assessment surveys’ data that were purposively collected in August 2013 and September 2015 from 40 health facilities that provide maternal and newborn services. These were thirty-three health center III’s, four health center IV’s and three hospitals. In addition, we purposively conducted 18 key informant interviews in September 2015 with the health workers in charge of maternity from 18 health facilities. To assess the demand side factors, we conducted 12 Focus Group Discussions (FGDs) in September 2015 with women who had delivered in the last 12 months and 12 FGDs with men whose wives had delivered in the last 12 months. These data were part of the data corpus for a maternal and newborn study that was conducted in Eastern Uganda [22].

### Data collection procedure, management, and analysis

#### Quantitative data

Three teams, each of which included the field supervisor and six research assistants were recruited and trained for 3 days on how to collect health facility assessment data. The research assistants were those who had completed the secondary school level. We developed the training data collection manual, which guided the training and the collection of data while in the field. Before data collection, supervisors contacted the health facility in-changes to be available and guide the research assistants on the day of data collection. A structured questionnaire was administered to the health facility worker and in some instances, observations were made to ascertain the availability and functionality of equipment and drugs. We collected data on staffing, days of reproductive service availability, and availability of lifesaving medicines and equipment.

Data were entered into an Excel database by a team of trained data entrants. Cleaning and consistency checks were conducted during data collection and entry. The excel files were then transferred to the SPSS v.20 for analysis. The data were mainly presented in the form of frequencies and percentages using tables and graphs.

#### Qualitative data

Three teams of trained research assistants each comprising of the supervisor, an interviewer, and note taker collected the qualitative data through voice recording and note-taking. The recordings were then transcribed verbatim and thereafter translated into English. The FGDs were limited to at most eight people, and these lasted for 1 hour. The FGDs were conducted in local languages and participants were given refreshments as well as a bar of soap (equivalent to 1.5USD) after the discussions. The KIs were conducted in English since these only involved the district leaders, community leaders, and health facility workers who were fluent in English speaking.

A team of three independent researchers, who had skills in qualitative data analysis analyzed the transcripts manually. Using deductive thematic analysis, the transcripts were reviewed and a set of codes developed to describe groups of categories. The grouped categories were then used to generate related study themes emerging from the data. The categories that emerged from transcripts were the lifesaving medicine availability, maternity space, skilled staff availability, transport system, access to information, poverty/financial accessibility, and the existence of traditional birth attendants. Given these categories, three themes emerged and these were the facility healthcare services, geographical/social/ economic factors and traditional birth attendants’ services. Direct quotations from health workers, women, and men are presented in italics to supports the findings.

## Results

### Facility health care (supply side) services

#### Availability of services

The availability of reproductive health services in the study health facilities are summarized in Table 1. Antenatal services, vaccination services, Prevention of Mother To Child Transmission (PMTCT) services, Postnatal care (PNC) services and maternity as well intrapartum services were provided in almost all health facilities at baseline and follow-up. However, abortion and kangaroo mother care services were not available in most of the health facilities at baseline and follow-up. Surprisingly, at follow-up, abortion services were not being provided at all health center IV and hospitals. At follow-up, quality assurance was being done in all hospitals and 3 out of 4 health center IVs.

**Table 1:** Availability of services

	*HC III* *n = 33*		*HC IV* *n = 4*		*Hospital* *n = 3*		*Total* *n* = 40	
	Baseline	Follow-up	Baseline	Follow-up	Baseline	Follow-up	Baseline	Follow-up
	*n(%)*	*n(%)*	*n(%)*	*n(%)*	*n(%)*	*n(%)*	*n(%)*	*n(%)*
Antenatal registration and counseling	33 (100.0)	33 (100.0)	3 (75.0)	4 (100.0)	3 (100.0)	3 (100.0)	39 (97.5)	40 (100.0)
Vaccination	32 (97.0)	32 (97.0)	3 (75.0)	4 (100.0)	3 (100.0)	3 (100.0)	38 (95.0)	39 (97.5)
PMTCT	33 (100.0)	32 (97.0)	3 (75.0)	4 (100.0)	3 (100.0)	3 (100.0)	39 (97.5)	39 (97.5)
Family planning counseling	33 (100.0)	31 (93.9)	3 (75.0)	4 (100.0)	2 (66.7)	3 (100.0)	38 (95.0)	38 (95.0)
Post-natal health checks for mother and newborn	32 (97.0)	28 (84.8)	3 (75.0)	4 (100.0)	3 (100.0)	3 (100.0)	38 (95.0)	35 (87.5)
PNC available	29 (87.9)	27 (81.8)	3 (75.0)	3 (75.0)	3 (100.0)	3 (100.0)	35 (87.5)	33 (82.5)
Maternity/ Intra-partum care	29 (87.9)	32 (97.0)	3 (75.0)	4 (100.0)	3 (100.0)	3 (100.0)	35 (87.5)	39 (97.5)
Abortion services	10 (30.3)	1 (3.0)	3 (75.0)	0 (0.0)	2 (66.7)	0 (0.0)	15 (37.5)	1 (2.5)
Quality assurance systems in place	25 (75.8)	13 (39.4)	3 (75.0)	3 (75.0)	2 (66.7)	3 (100.0)	30 (75.0)	19 (47.5)
Kangaroo Mother Care practice	9 (27.3)	22 (66.7)	2 (50.0)	3 (75.0)	1 (33.3)	1 (33.3)	12 (30.0)	26 (65.0)

#### Availability of protocols

Table 2 summarizes the availability of protocols and health promotion materials found at the health facilities. Availability of health facility staff organogram and policy for needle stick injury of patients or staff increased by at least 40%. Protocol for major hemorrhage and partograph use increased by 37 and 50%, respectively. However, at follow-up, they were still missing in at least 30% of the health facilities. In addition, at baseline and follow-up, at least 30% of the health facilities did not have promotion/educative messages on family planning use and HIV/STI preventions.

**Table 2:** Availability of protocols and education materials

	*HC III* *n = 33*		*HC IV* *n = 4*		*Hospital* *n = 3*		*Total* *n* = 40	
	Baseline	Follow-up	Baseline	Follow-up	Baseline	Follow-up	Baseline	Follow-up
	*n(%)*	*n(%)*	*n(%)*	*n(%)*	*n(%)*	*n(%)*	*n(%)*	*n(%)*
Organogram of the health facility staff	6 (18.2)	18 (54.5)	1 (25.0)	3 (75.0)	1 (33.3)	2 (66.7)	8 (20.0)	23 (57.5)
Policy for needle stick injury of patients or staff	8 (24.2)	18 (54.5)	1 (25.0)	3 (75.0)	1 (33.3)	3 (100.0)	10 (25.0)	24 (60.0)
Policy for disposal of sharp equipment	25 (75.8)	32 (97.0)	3 (75.0)	4 (100.0)	3 (100.0)	3 (100.0)	31 (77.5)	39 (97.5)
Protocol for referral	9 (27.3)	22 (66.7)	0 (0.0)	4 (100.0)	1 (33.3)	2 (66.7)	10 (25.0)	28 (70.0)
Protocol for major haemorrhage	5 (15.2)	14 (42.4)	0 (0.0)	4 (100.0)	1 (33.3)	3 (100.0)	6 (15.0)	21 (52.5)
Protocol for use of partograph	6 (18.2)	23 (69.7)	1 (25.0)	4 (75.0)	1 (33.3)	2 (66.7)	8 (20.0)	28 (70.0)
Poster/leaflets promoting prevention services	25 (75.8)	24 (72.7)	1 (25.0)	4 (75.0)	1 (33.3)	0 (0.0)	27 (67.5)	27 (67.5)
Posters promoting family planning	25 (75.8)	24 (72.7)	2 (50.0)	4 (100.0)	1 (33.3)	0 (0.0)	28 (70.0)	28 (70.0)
Poster promoting nutrition	13 (39.4)	23 (69.7)	2 (50.0)	3 (75.0)	2 (66.7)	2 (66.7)	17 (42.5)	28 (70.0)

#### Availability of lifesaving drugs and equipment

The availability of quality maternal and newborn services depends greatly on the availability of essential equipment, drugs, health facility space, and qualified health workers. The facility assessment survey indicated that most health facilities including hospital and health center IVs persistently had no disposable gloves, stethoscope, fetal scope, Manual vacuum aspirator for abortion care, aspiration kit, oxygen, blank partograph, and vacuum extractor in the two surveys (Table 3). In addition, blankets to wrap the newborn, baby warmer and binders for kangaroo mother care were lacking in most of the health facilities at baseline and follow-up (Table 3). Other essential equipment such as blood pressure machines, sterile scissor, and working watch were not available in at least 20% of the health facilities in both surveys (Table 3).

**Table 3:** Availability of essential equipment

	*HC III* *n = 33*		*HC IV* *n = 4*		*Hospital* *n = 3*		*Total* *n* = 40	
	Baseline	Follow-up	Baseline	Follow-up	Baseline	Follow-up	Baseline	Follow-up
	*n(%)*	*n(%)*	*n(%)*	*n(%)*	*n(%)*	*n(%)*	*n(%)*	*n(%)*
General equipment								
Child health record/vaccination cards	14 (42.0)	19 (57.6)	1 (25.0)	3 (75.0)	1 (33.3)	2 (66.7)	16 (40.0)	24 (60.0)
Blood pressure machine (sphygmomanometer)	18 (55.0)	25 (75.8)	3 (75.0)	4 (100.0)	2 (66.7)	3 (100.0)	23 (57.5)	32 (80.0)
Sterile scissors or blade	21 (64.0)	22 (66.7)	3 (75.0)	4 (100.0)	2 (66.7)	3 (100.0)	26 (65.0)	29 (72.5)
Working adult scale	18 (55.0)	29 (87.9)	3 (75.0)	4 (100.0)	3 (100)	3 (100.0)	24 (60.0)	36 (90.0)
Working watch or timing device	16 (48.0)	20 (60.6)	3 (75.0)	4 (100.0)	2 (66.7)	2 (66.7)	21 (52.5)	26 (65.0)
Room with visual privacy	29 (88.0)	26 (78.8)	3 (75.0)	3 (75.0)	3 (100)	3 (100.0)	35 (87.5)	32 (80.0)
24- h functioning light source	17 (52.0)	20 (60.6)	2 (50.0)	4 (100.0)	3 (100)	3 (100.0)	22 (55.0)	27 (67.5)
Maternal and newborn equipment								
Disposable gloves	30 (91.0)	26 (78.8)	1 (25.0)	3 (75.0)	3 (100)	2 (66.7)	34 (85.0)	31 (77.5)
Stethoscope	22 (67.0)	26 (78.8)	3 (75.0)	3 (75.0)	2 (66.7)	2 (66.7)	27 (67.5)	31 (77.5)
Fetal scope	32 (97.0)	27 (81.8)	3 (75.0)	3 (75.0)	2 (66.7)	2 (66.7)	37 (92.5)	32 (80.0)
Working baby scale	23 (70.0)	24 (72.7)	2 (50.0)	3 (75.0)	3 (100.0)	3 (100.0)	28 (70.0)	30 (75.0)
Thermometer	14 (42.0)	20 (60.6)	3 (75.0)	3 (75.0)	3 (100.0)	3 (100.0)	20 (50.0)	27 (67.5)
Intravenous fluids with infusion set	23 (70.0)	27 (81.8)	3 (75.0)	3 (75.0)	3 (100.0)	3 (100.0)	29 (72.5)	33 (82.5)
Manual vacuum aspirator for abortion care	7 (21.0)	15 (45.5)	3 (75.0)	3 (75.0)	1 (33.3)	2 (66.7)	11 (27.5)	20 (50.0)
Speculum	19 (58.0)	23 (69.7)	2 (50.0)	3 (75.0)	2 (66.7)	3 (100.0)	23 (57.5)	29 (72.5)
Aspiration kit	4 (12.0)	11 (33.3)	1 (25.0)	2 (50.0)	0 (0.0)	2 (66.7)	5 (12.5)	15 (37.5)
Oxygen	6 (18.0)	6 (18.2)	1 (25.0)	3 (75.0)	1 (33.3)	2 (66.7)	9 (22.5)	11 (27.5)
Blank partographs	7 (21.0)	17 (51.5)	1 (25.0)	3 (75.0)	1 (33.3)	2 (66.7)	10 (25.0)	22 (55.0)
Vacuum extractor (for vacuum delivery/assisted delivery)	1 (3.0)	9 (27.3)	2 (50.0)	2 (50.0)	1 (33.3)	2 (66.7)	5 (12.5)	13 (32.5)
Newborn suction device	8 (24.0)	12 (36.4)	1 (25.0)	3 (75.0)	3 (100.0)	3 (100.0)	12 (30.0)	18 (45.0)
Newborn resuscitation device/Ambu bag/Mask	17 (52.0)	19 (57.6)	3 (75.0)	3 (75.0)	3 (33.3)	3 (100.0)	21 (52.5)	25 (62.5)
Clamp or umbilical tie	25 (76.0)	25 (75.8)	3 (75.0)	4 (100.0)	2 (66.7)	3 (100.0)	30 (75.0)	32 (80.0)
Gentian violet paint	9 (27.0)	7 (21.2)	1 (25.0)	1 (25.0)	2 (66.7)	1 (33.3)	12 (30.0)	9 (22.5)
Dextrose saline/ORS	20 (61.0)	25 (75.8)	2 (50.0)	4 (100.0)	3 (100.0)	3 (100.0)	25 (62.5)	32 (80.0)
Nasogastric tubes 20 ml syringes	6 (18.0)	5 (15.2)	2 (50.0)	2 (50.0)	1 (33.3)	2 (66.7)	9 (22.5)	9 (22.5)
Binders for Kangaroo Mother Care	1 (3.0)	3 (9.1)	0 (0.0)	1 (25.0)	0 (0.0)	0 (0.0)	1 (2.5)	4 (10.0)
Blanket to wrap newborn	1 (3.0)	6 (18.2)	0 (0.0)	0 (0.0)	0 (0.0)	1 (33.3)	1 (2.5)	7 (17.5)
Baby warmer or heat lamp	0 (0.0)	1 (3.0)	0 (0.0)	1 (25.0)	0 (0.0)	1 (33.3)	1 (2.5)	3 (7.5)

Additionally, pregnancy test kits, proteinuria, and anemia tests were persistently not available in most of the health facilities including some health center IV’s and hospitals at both baseline and follow-up (Table 4). There was an improvement in the stock of malaria, HIV rapid, and syphilis test kits, though there were out of stock in some facilities.

**Table 4:** Availability of testing kits

	*HC III* *n = 33*		*HC IV* *n = 4*		*Hospital* *n = 3*		*Total* *n* = 40	
	Baseline	Follow-up	Baseline	Follow-up	Baseline	Follow-up	Baseline	Follow-up
	*n(%)*	*n(%)*	*n(%)*	*n(%)*	*n(%)*	*n(%)*	*n(%)*	*n(%)*
Pregnancy test kits	11 (33.3)	22 (66.7)	3 (75.0)	3 (75.0)	1 (33.3)	0 (0.0)	15 (37.5)	25 (62.5)
Proteinuria	16 (48.5)	24 (72.7)	1 (25.0)	3 (75.0)	2 (66.7)	0 (0.0)	19 (47.5)	27 (67.5)
Rapid test for malaria	19 (57.6)	32 (97.0)	3 (75.0)	4 (100.0)	3 (100.0)	0 (0.0)	25 (62.5)	36 (90.0)
HIV rapid tests	21 (63.6)	30 (90.9)	1 (25.0)	4 (100.0)	3 (100.0)	3 (100.0)	25 (62.5)	37 (92.5)
Syphilis RPR syphilis tests	4 (12.1)	27 (81.8)	2 (50.0)	4 (100.0)	1 (33.3)	3 (100.0)	7 (17.5)	34 (85.0)
Syphilis rapid tests	4 (12.1)	27 (81.8)	3 (75.0)	4 (100.0)	1 (33.3)	3 (100.0)	8 (20)	34 (85.0)
Anaemia tests	9 (27.3)	16 (48.5)	4 (100.0)	2 (50.0)	2 (66.7)	2 (66.7)	15 (37.5)	20 (50.0)

Regarding lifesaving medicines, overall, most of the health facilities did not have essential drugs for maternal and newborn health at baseline and follow-up (Table 5). Drugs such as ferrous sulphate, folic acid, combined ferrous, benzyl-penicillin, diazepam, mebendazole, amoxicillin, and penicillin were not available in at least 30% of the health facilities. Oxytocin, misoprostol, ergometrine, and corticosteroids were not available in all health facilities in both surveys. Additionally, IV metronidazole, IV gentamycin, IV ampicillin, magnesium sulphate, zinc tablets, and nevirapine, were persistently not available in most of the health facilities. Surprisingly, misoprostol, ergometrine, corticosteroid, IV gentamycin, nevirapine, and magnesium sulphate (IV or IM) were also not available in some hospitals and health center IV at baseline and follow-up.

**Table 5:** Availability of drugs

	*HC III* *n = 33*		*HC IV* *n = 4*		*Hospital* *n = 3*		*Total* *n* = 40	
	Baseline	Follow-up	Baseline	Follow-up	Baseline	Follow-up	Baseline	Follow-up
	*n(%)*	*n(%)*	*n(%)*	*n(%)*	*n(%)*	*n(%)*	*n(%)*	*n(%)*
Sulphadoxine Pyrimethamine for IPTp	27 (81.8)	30 (90.9)	4 (100.0)	2 (50.0)	1 (33.3)	3 (100.0)	32 (80.0)	35 (87.5)
Vitamin A	30 (90.9)	31 (93.9)	4 (100.0)	3 (75.0)	3 (100)	3 (100.0)	37 (92.5)	37 (92.5)
Ferrous Sulphate	14 (42.4)	19 (57.6)	3 (75.0)	2 (50.0)	2 (66.7)	2 (66.7)	19 (47.5)	23 (57.5)
Folic Acid	16 (48.5)	23 (69.7)	2 (50.0)	2 (50.0)	2 (66.7)	2 (66.7)	20 (50.0)	27 (67.5)
Combined ferrous/folate	9 (27.3)	19 (57.6)	4 (100.0)	2 (50.0)	2 (66.7)	2 (66.7)	15 (37.5)	23 (57.5)
Benzyl penicillin	15 (45.5)	13 (39.4)	2 (50.0)	2 (50.0)	3 (100)	3 (100.0)	20 (50.0)	18 (45.0)
Diazepam (IM or IV)	28 (84.8)	15 (45.5)	2 (50.0)	4 (100.0)	3 (100)	3 (100.0)	33 (82.5)	22 (55.0)
Mebendazole	29 (87.9)	19 (57.6)	4 (100.0)	3 (75.0)	3 (100)	2 (66.7)	36 (90.0)	24 (60.0)
Amoxicillin	12 (36.4)	21 (63.6)	1 (25.0)	3 (75.0)	1 (33.3)	2 (66.7)	14 (35.0)	26 (65.0)
Penicilin or ampicillin	10 (30.3)	21 (63.6)	3 (75.0)	3 (75.0)	1 (33.3)	2 (66.7)	14 (35.0)	26 (65.0)
Cotrimoxizole	26 (78.8)	28 (84.8)	3 (75.0)	4 (100.0)	3 (100)	3 (100.0)	32 (80.0)	35 (87.5)
Tetracycline ointment or silver nitrate eye drops	20 (60.6)	26 (78.8)	2 (50.0)	4 (100.0)	2 (66.7)	2 (66.7)	24 (60.0)	32 (80.0)
Corticosteroids (for preterm labour)	8 (24.2)	10 (30.3)	1 (25.0)	1 (25.0)	1 (33.3)	1 (33.3)	10 (25.0)	12 (30.0)
Ergometrine (oral or injectable)	3 (9.1)	5 (15.2)	2 (50.0)	1 (25.0)	1 (33.3)	1 (33.3)	6 (15.0)	7 (17.5)
Oxytocin	10 (30.3)	27 (81.8)	3 (75.0)	4 (100.0)	3 (100)	2 (66.7)	16 (40.0)	33 (82.5)
Misoprostol	8 (24.2)	2 (6.1)	1 (25.0)	3 (75.0)	2 (66.7)	1 (33.3)	11 (27.5)	6 (15.0)
IV Ampicillin	9 (27.3)	3 (9.1)	2 (50.0)	1 (25.0)	1 (33.3)	2 (66.7)	12 (30.0)	6 (15.0)
IV Gentamycin	8 (24.2)	1 (3.0)	1 (25.0)	2 (50.0)	2 (66.7)	1 (33.3)	11 (27.5)	4 (10.0)
IV Metronidazole	5 (15.2)	0.0	1 (25.0)	2 (50.0)	1 (33.3)	2 (66.7)	7 (17.5)	4 (10.0)
Local anaesthetics (such as lidocaine)	22 (66.7)	4 (12.1)	2 (50.0)	4 (100.0)	3 (100)	2 (66.7)	27 (67.5)	10 (25.0)
Zinc tablets	20 (60.6)	4 (12.1)	2 (50.0)	2 (50.0)	2 (66.7)	2 (66.7)	24 (60.0)	8 (20.0)
Nevirapine	21 (63.6)	3 (9.1)	2 (50.0)	4 (100.0)	3 (100)	1 (33.3)	26 (65.0)	8 (20.0)
Magnesium Sulphate (IV or IM)	9 (27.3)	2 (6.1)	(0.0)	2 (50.0)	2 (66.7)	1 (33.3)	11 (27.5)	5 (12.5)

The qualitative information from the health workers confirms the resource deficiencies and how these affect their work. These deficiencies ranged from basic sundries to emergency drugs and equipment. Often when there is stock out of drugs, women are requested to buy them from the drug shops. However, during the night accessing these supplies is impossible.

*“First and foremost some supplies such as gloves are always missing, and these things hinder mothers from coming to us because we ask them for three pairs of gloves. One pair is for the first examination, the second pair for the second examination, and the third for handling delivery and each pair is 1000/= it is too much for the mothers. You find that they give us 100 or 200 pairs of gloves and they are supplied to the center even in the outpatient department they need it and here in maternity, we need it so the supply is really low. Even the drugs are in most cases in shortage, for example, oxytocin, it is the same with magnesium for mothers with Pre Eclamptic Toxemia (PET).”* Health center III, Kamuli district.

*“............ the challenge we are having now is that there is always stock out of drugs and other essential equipment needed for maternal and newborn care. For example, we have not been having syphilis reagents for last 12 months. We also don’t have newborn equipment like bulb syringes for newborn resuscitation”* HC III, Kibuku district.

Surprisingly, the higher-level facilities that are intended to be referral centers also experience inadequate emergency supplies, which deprive women to further access quality services as pointed out by one the health worker during KI.

*“……now sometimes if a mother comes when she in* antepartum hemorrhage (*APH), sometimes we don’t have blood so by the time when you refer her sometimes she can reach there when she is in a very critical condition.”* Health center IV, Kamuli district.

#### Referral transport

In addition, the KIs confirmed the lack of referral transport systems in most of the health facilities. Yet the availability of transport is very critical in referring women to the higher-level health facilities for further management. As indicated by some of the health workers during KIs, often, facilities improvise by using ‘boda-boda’ motorcycles, which are not safe for transporting mothers in critical conditions. Women regularly fail to get the transport more especially during night hours, which delays their referral to the higher-level health facilities.

***“*** *We have a challenge of transportation of mothers. A mother may come here with complication and you need to refer her to hospital. From here we have that small ambulance (that tri-cycle), but it is not readily available you may call him [transporter] when he is 20 kilometers away and yet you have an emergency. Because of that, you may end up taking [transporting] this mother on a motorcycle to Kamuli [hospital]. So, referral system is still a challenge.”* Health center III, Kamuli district.

*“One problem is transport, sometimes the doctor might not be there or an anesthesia nurse might not be there so you want to refer that patient to another hospital but there is no transport. For example, we had a lady who was transferred from a clinic and we had no transport, that lady’s husband had to go and look for public means and he failed until morning when we went to Pallisa [town]. So, suppose we had that delivery I think that woman would have died, but God helped us and we got transport in the morning and they went and the mother was operated from there [regional hospital].”* Hospital, Pallisa district.

#### Space in Maternity Wards

Space in maternity wards was also indicated to be inadequate which in most cases leads to the discharge of newly delivered women before the recommended postpartum time. Due to the limited space in the maternity wards, sometimes deliveries are conducted on the floor.

***“*** *We have very small space as you can see. Labor ward has only two beds. Last night I had three mothers and one had to deliver from the floor due to the limited number of the beds here. For example, one mother came during the day, she was in false labor, and we kept her because we could not tell her to go home because she had signs that maybe she could start the true labor. Another one came she was in real labor, we admitted her, then the third one came, she was also in true labor we also admitted. Then it was around 1:00 Am in the night and on examining them, they were all fully in labor, remember we have only two beds, so one had to deliver from the floor!”* Health center III, Pallisa district.

*“We don’t have enough space for delivering mothers. When you get another delivering mother you will not have where to put her. Because of that, you will have to discharge the first one who has just delivered; yet, you should monitor this mother who has delivered for at least 12 hours. But, because they are many, they are uncomfortable; the room is too small they request for discharge to go home.”* Health center III, Kibuku district.

#### Availability of qualified staff

Table 6 indicates the number of health workers available at the health facilities. From the baseline and follow-up facility assessment surveys, employment of clinicians, midwives, enrolled nurses and registered nurses increased by 50%, 11%, 20% and 47%, respectively. However, the availability of midwives is still below 50% (Table 4).

**Table 6:** Availability of health workers

	Baseline		Follow-up	
	Number	Percent	Number	Percent
Overall	40	100.0	40	100.0
Registered midwives				
None	31	77.5	23	57.5
One	6	15.0	10	25.0
Two	3	7.5	3	7.5
at least three	0	0.0	4	10.0
Enrolled Nurse				
None	12	30.0	4	10.0
One	13	32.5	2	5.0
Two	8	20.0	6	15.0
At least three	7	17.5	28	70.0
Clinician				
None	25	62.5	5	12.5
One	11	27.5	13	32.5
Two	2	5.0	16	40.0
At least three	2	5.0	6	15.0
Registered Nurse				
None	28	70.0	9	22.5
One	9	22.5	22	55.0
Two	3	7.5	6	15.0
At least three	0	0.0	3	7.5
Nursing Assistants				
None	16	40.0	3	7.5
One	15	37.5	15	37.5
Two	6	15.0	10	25.0
At least three	3	7.5	12	30.0

The KIs with the health workers confirm the inadequacy of skilled health workers responsible for providing maternal and newborn health. Because most of the health facilities have one midwife, they usually work during the day and night without having rest hours. Normally, one midwife at the facility does all maternal and child health work raging from ANC, delivery, postnatal care to immunization 24/7 (throughout the week).

*“...you see the community think we are rude, but it is the nature of our work. For example, at this facility, we are only two health workers (in-charge and midwife). The midwife works day and night. There is a lot of work. Seeing pregnant women who have come for ANC, Immunization, and deliveries by one person without rest! ... hmmm... We are also human beings who need rest.”* Health center III*,* Pallisa district.

***“…*** *understaffing is a big challenge. If we were many, you cannot work at night and throughout the day. By now I would be at home resting, waiting for night duty but now I am here because I cannot leave those mothers unattended to. Sometimes you are not on time, for example, when I went after night duty; I first had to go home in the morning to freshen up and also take some breakfast before coming back here. By the time you come back you find that these mothers have sat for long hours waiting for you here, which is a very big problem”* Health center III, Kamuli district.

**Fig. 1:**
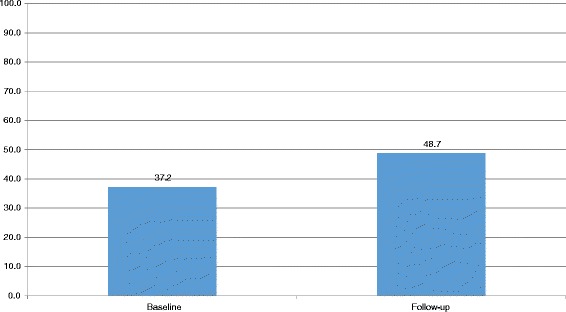
Facility that reported use of Nursing Assistants in conducting deliveries

The challenge of increased utilization of services amidst inadequate staffing also drives facilities towards using unqualified nursing assistants. For instance, the health facilities improvise by using the nursing assistants who are not qualified to conduct deliveries in the absence of a midwife. During the health facility assessment, participants were asked to tell if they use nursing assistants to conduct deliveries and it was revealed that around 50% of the health facilities use nursing assistants to conduct deliveries (Fig. 1).

During key informant interview, the health workers also confirmed the use of nursing assistants in conducting assisting women to deliver at health facilities.

*“............the challenge we are having now is that the facility does not have the midwife. We are improvising by using the Nursing Assistant who is not trained to do that work.* **”** HC III staff, Pallisa.

*“The first challenge we have is that we lack enough health workers. For example, in this facility, the number of clients has increased but I am the only midwife who works on this increased number. Because of that, we end up delegating to the nursing assistants who we always work with to cover us in case we are not available at the health center.”* Health center III, Kibuku district.

### Demand-side influences on maternal and newborn care

The themes that emerged through FGDs, which hinder the utilization of maternal services, were health facility services, geographical/social/economic factors and traditional birth attendants’ services.

### Facility health care services

The persistent supply-side bottlenecks such as staff absenteeism and inadequate drugs and equipment impede the accessibility/utilization of maternal and newborn care services. The interviews with the community indicated how for instance absenteeism of the health workers, health workers’ attitude and skills affect health facility delivery. In some facilities, health workers are there on specific days, meaning that the services are not available throughout the week (7 days a week).

*“……. we have a problem in this community, health workers are only available on Monday, Tuesday and Wednesday. The other remaining days of the week, when you go you will not find the health workers and yet you are in labor. So, we end up delivering from home since we know that most of the times the health workers are not always there.”* Women FGD kibuku district.

*“The health workers at our facilities are rude and they always neglect us. The Traditional Birth Attendants have good care other than going to the government hospitals and suffer from there.”* FGD women Kamuli district.

### Geographical, social and economic factors

The geographical, social and economic factors included transport systems including distance to the health facilities, access to information, poverty, and access to finances.

#### Transport system

Long distances were indicated to be affecting women from delivering at the health facilities, which affects even those who would wish to deliver at the health facilities. Therefore, women end-up delivering at traditional birth attendants since they are closer to them.

“*Most women deliver from home like he has said and what brings that is the long distance (about 8–10 Kms to the health facilities).”* Female FGD Pallisa district.

“*The reason as to why women are delivering from home is because of the long distance to the health facilities. If a woman starts experiencing labor pains, it might be hard for the man to get transport at that time and maybe the time he gets a bicycle the woman might have reached a stage that she cannot even sit on the bicycle. So, at the end, they have to pick those old women and that is still happening in this place; those who have ever conducted deliveries to come and help to deliver.*” FGD Male, Pallisa district.

“………. *very few women who deliver from the facility, though many would wish to deliver from the facility but because of the long distance……… those who try to go and deliver from the health facility at times end up delivering on the way because of the long distance that they move and if people who attend to her at that don’t have the skills to deliver a woman, then the baby can even die from there.”* Female FGD, Kibuku district.

Besides long distances to the health facilities, the FGD participants pointed out a problem of transport in these rural communities, resulting from poor roads and high transport charges.

*“….the challenge is that sometimes they [women] do not reach there [health facility] on time due to the poor transportation system in this village and the poor roads. So, by the time they reach, the babies are at a high risk of dying.”* FGD men, Kamuli district.

*“The main difficulty that we mothers face when we are going to deliver is the long distance to the Health Centers and the poor transport system. So, by the time the mothers reach, they are usually in critical conditions.”* FGD women, Kamuli district.

*“Distance and lack of transport to take you to the health facility are some of the reasons why women give birth at home. Also, labor pains may start during the night and sometimes it might have rained, which is so hard for you to reach a health facility. So, if you get someone to help you deliver the baby then someone would prefer delivering at home than risking going to the hospital.”* FGD women, Kibuku.

#### Access to information

The community members also pointed out lack of access to communication/sensitization as one the factors that lead to poor utilization of maternal services and as a result, women in these rural communities have not yet appreciated the importance of giving birth under the assistance of skilled health provider. In these communities, the health facilities rarely do community outreaches to sensitize the community.

*“…people in this community have not yet realized the importance of delivering from the health facility. So there is limited information on skilled deliveries.”* FGD men, Kamuli district.

*“People lack knowledge and information on safe, skilled deliveries and need to be healthy educated.”* FGD women, Kamuli district.

*“…. The biggest challenge is that there are no outreaches that come from the health facilities to health educate our women and reduce maternal and newborn deaths. So, you find that the number of maternal and newborn deaths is just increasing instead of reducing due to lack of knowledge. So, it would be good if only the Government came up with a program to health educate people about the many risks of delivering from home and also bringing services nearer to the people.”* FGD men, Pallisa district.

#### Poverty and access to finances

Both health workers and community indicated poverty and lack of finances to pay at the health facilities as one of the factors that stop some women from seeking maternal and newborn services. Low incomes and long distances to the health facilities that are associated with high transport costs frequently force women to delivery at home or with TBAs. In some instances when the women are referred to the high-level health facilities, they do not honor the referrals because of costs related to transport. Too often, women risk giving birth at lower level health centers and home or TBAs homes, regardless of their awareness about the complications.

“*The health center that we have around is 5 kms away and it is private so services in that facility are expensive. Right now, if you take a woman to deliver from there and then she gives birth to a baby boy they will charge you 40,000/− and if it is a baby girl, you pay 50,000/− that is why when a woman starts getting labor pains a person starts pretending until the woman delivers from home.” FGD Men Pallisa.*

*“Sometimes you may get a mother who comes with complications and yet during ANC, you told her that due to for instance, certain complications, she will not deliver from here (Health center III) but because the mother does not have money, she comes back only to check on her passport book you had referred her (to the hospital) but the mother insisted on coming back because she did not have the money to go to where you referred her. So, we end up paying the transport for such a mother because the mother may end up dying from here and you are the one to be blamed.*” Health facility III, Kamuli district.

*“What stops us from going to other medical centers is because of our low incomes, the poor transport. We usually use boda-bodas (motorcycles) which usually charge expensively for example, they may charge us around 20,000/= for hire of their motorcycle because they know you are in need and don’t have many options, hence they hike the charges when they know that we don’t have any other option but to use them.”* FGD women, Kibuku district.

### Traditional birth attendants’ services

The poor services at the health facilities and other factors such as poor community transport system that lead to high costs of transport, promote Traditional Birth Attendants (TBAs) deliveries as the best alternative. In fact, some communities consider (TBAs) to be better and cheaper, considering what it takes to deliver at the health facility. Sometimes, women who go to deliver at the health facilities deliver on their own with no assistance from a health worker, which demotivates other since they feel it does not make any difference from a delivery conducted at home or TBA.

*“Someone would desire to reach those other Health Centre’s but because of the poor transport and the bad roads, they chose to opt for the nearby services even when they are inadequate and the mothers’ lives are unsafe.”* FGD Women, Pallisa district.

*“… she (TBA) was near my home and so I decided to use her than going to the health center.”* FGD women, Pallisa district.

*“The TBAs have good care other than going to the government hospitals and suffer from there. Also, the distance from your home to the TBAs may be near so you save money for transport and get good care from her.”* FGD women, Kamuli district.

*“We give birth at Ruth’s, Adiye and Kayendeke homes (TBAs). They are not trained, but the community trusts them and it is the reason why we prefer delivering from there.”* Women FGD, Pallisa district.

*“When the time for delivery reaches, we call the TBA to help because we will not spend much, and if we have to give anything, it could be just a small chicken and things end there. So, they [women] deliver from TBAs because there is not much disturbance than it would be if they are to deliver from the health facility.”* FGB men Kibuku district.

### Summarizing the interrelationship between demand and supply factors for MNH

**Fig. 2:**
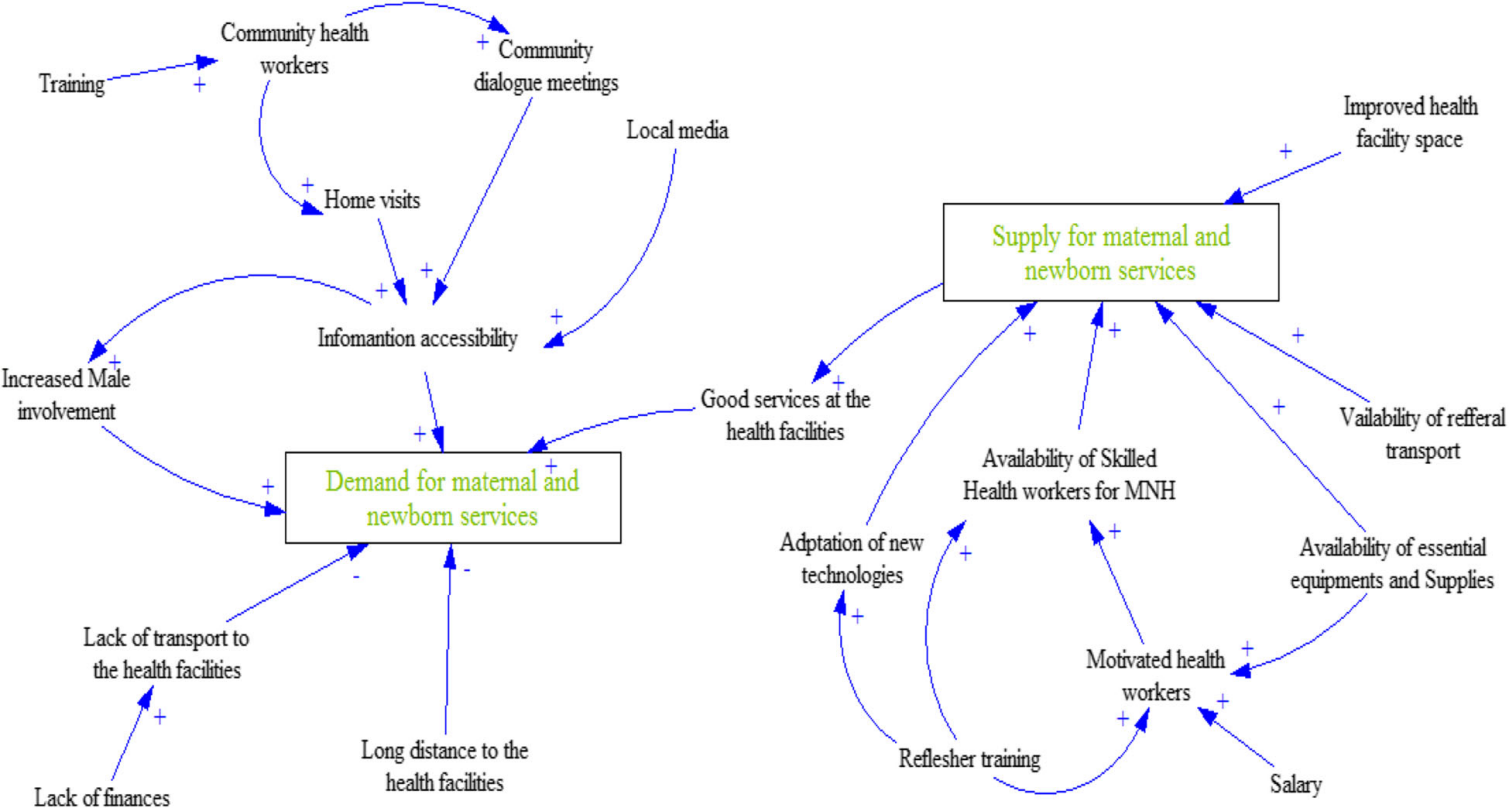
Causal loop diagram summarizing demand and supply side interactions for maternal and newborn services

Figure 2 is a causal loop diagram that summaries how persistent supply side and demand side gaps interact to constrain progress in achieving of maternal and newborn outcomes in rural Uganda. The figure indicates how the availability of services such as health workers, drugs, and use of technologies among others are all crucial for the provision of quality service, which in turn motivate the community to access the facility services, thereby promoting the demand for maternal and newborn care services. However, there are other community (both structural and cultural) factors constrain mothers from accessing the health facility services.

## Discussion

The findings of this study confirm the persisting demand and supply factors that might have contributed to the sluggish progress in reducing maternal and newborn death in Uganda. In addition, this paper explains the reasons for existing variation in accessing the quality of maternal and newborn care in the country. This paper, therefore, provided plausible reasons why Uganda and perhaps other LMICs continue to register unacceptably high maternal and newborn deaths compared to developed countries. The study indicates the importance of addressing both demand and supply side factors if countries, particularly LMICs are to significantly reduce maternal and newborn deaths. In the subsequent subsections, we discuss the supply and demand side factors and how they are interrelated.

### Supply-side factors

This study confirmed the persisting lack of lifesaving commodities in most of the rural health facilities including high-level referral health facilities. Essential drugs such as parenteral antibiotics drugs; uteroteonic drugs such as oxytocin, parenteral anticonvulsants for preeclampsia and eclampsia such as magnesium sulfate were missing in most of the health facilities in both years of data collection. In addition, essential equipment such as those needed to manually remove the placenta and retained products (for example, manual vacuum extraction, dilation and curettage), those that are used to perform assisted vaginal delivery (for instance Vacuum extraction and forceps delivery) and basic neonatal resuscitation (for instance with ambu bag and mask) items were missing in most of the health facilities in both years of data collection. Surprising, most of these essential medicines and equipment were missing in some of the hospital and health center IVs, which are referral health facilities that are responsible for handling comprehensive EmoC cases. Besides, these supplies have been indicated as the signal functions used to identify basic and comprehensive emergency obstetric care services [26–28]. The supplies are crucial for the management of hemorrhage, prolonged or obstructed labor, postpartum sepsis, pre-eclampsia or eclampsia, ectopic pregnancy, ruptured uterus and newborn distress (intrapartum) [26], which are major causes of maternal and newborn death. One of the reasons for inadequate drugs and equipment in most of the rural health facilities result from the weak supply chain, inadequate regulatory capacity and little knowledge of where to get the supplies from [16]. Strengthening the supply chain and improving the health workers’ skills in proper requisitioning for essential medicines is thus crucial. In Uganda, the National Medical Stores (NMS) is a body authorized by the government to deliver medicine and equipment to the public health facilities. However, too often, the distribution of these supplies is not based on the facility population coverage or utilization rate and in some instances, lower level health facilities receive unnecessary equipment/drugs that do not fit their mandate. The district health management, health facility managers, and NMS should work together to ensure that appropriate drugs and equipment are requested and delivered on time.

Interventions to manage low birth weight babies were missing in most of the health facilities in both years of data collection. Less than a fifth of health facilities indicated having Kangaroo mother care services such as blanket to wrap the baby, baby warmer, and binders for kangaroo mother care. Surprisingly, these were also missing in most of the hospitals and health center IVs. This does not surprise why most of the low birth weight babies in such settings do not survive [29]. In Uganda, it is estimated that around 10% of newborns are low birth babies [10] and this might be underestimated since most of the newborns are not always assessed for low birth weight [30]. These results call for additional emphasis on low birth weight identification and survival intervention. Fortunately, there are low-cost effective interventions such as foot length for the identification of low birth weight [31–33] and Kangaroo mother care services that increases the survival of low birth weight or preterm babies [29, 34] that can be replicated in these rural communities.

Further, the use of the partograph and active management of the third stage of labor were missing in most of the health facilities. Yet these are moral obstetric practice that should be used for all women in labor to prevent prolonged and obstructed labor [26], which helps to prevent uterine rupture and birth asphyxia that are among the leading causes of maternal and newborn mortality, respectively. Additionally, the use of partograph helps the health care providers to make timely decisions [35]. However, the use of partograph is still a challenge in most of the low and income countries as highlighted by other researchers [35]. Interventions for translating implementation clinical guideline to the health workers such as continuous mentorship and support supervisions that has been revealed as an effective intervention in strengthening the skills of health workers should be put in place.

In addition, the health facilities lacked skilled health workers responsible for maternal and newborn services. According to WHO, a skilled health worker responsible for maternal and newborn health is someone who has core midwifery skills and for the health worker working in the rural areas, they should be able to use vacuum extraction, manual vacuum aspiration, and management of obstructed labor [36]. However, in this study, at least a half of the health facilities did not have a trained midwife, who is responsible for providing maternal and newborn services. The use of unqualified nursing assistants to conduct deliveries may be no better than a traditional birth attended delivery because it puts women at greater risk amidst lack of drugs, supplies and referral transport. Such inadequacies in skilled health workers, equipment, and drugs compromise the quality of care [21, 37–40]. Unfortunately, this study did not collect information on the availability of other important cadres at high-level health facilities such as obstetrician-gynecologist. However, the absence of these cadres is always expected in rural health facilities as documented by other researchers [41].

### Demand-side factors

The lack of health workers, equipment, and drugs in health facilities do not only compromise the quality of care but also demoralize the community from seeking maternal and newborn services from health facilities [21, 22, 37–40]. In situations when women get to know that the facilities do not have drugs, equipment, and health workers, they may end-up accessing services from communities (private clinics and traditional birth attendants) or remain at home from the time they become pregnant to the time of delivery and may only go to the health facilities when complications have emerged [41].

The lack of reliable and affordable transport system between the health facility and community level was indicated as a critical player in the delivery of and access to timely health services [21, 42]. The dyad of long distance and poor transport system to the health facilities remains a major obstacle for seeking health facilities in sub-Saharan Africa [21, 37, 43–45]. The transport and road infrastructure act as a key link between potential accessibility and actual utilization of health services [21, 42]. In addition, transport enables timely transfer of patients between health facilities and facilitates easy referral between the different levels of care [42]. This, therefore, means that poor road infrastructure, as well as lack of transport at the community level, can influence the communities to access health care from less trained providers who are in most cases closer to them [42, 44]. Evidence from other studies has indicated how proximity to the health facilities influences health facility utilization [37, 44]. The poor transport system is also linked to the high cost of transport services to the health facilities [21, 42], which was also pointed out in this study. To address this problem, the communities should be supported to set-up sustainable and reliable mechanisms [42], such as organizing the communities to save for maternal and newborn health or to engage in other conditional income generating schemes.

Additionally, poverty and lack of money to pay for the service cost of the health facilities was also another factor mentioned. This is consistent with one of the studies done in African countries [37]. It is worth noting that the cost of services at the health facilities influences the mother’s service choice [21, 43, 44]. However, women from poorer families who are the majority in these rural communities are less likely to utilize health services compared to those belonging to the less poor families [21]. This, therefore, calls for innovative mechanisms for improving household income and savings for health in these communities. There is a need to learn from community health insurance interventions that have been implemented in other countries to see if they can be replicated in Uganda’s setting.

Furthermore, the communities also indicated lack of information as an obstacle for utilization of maternal and newborn services. This is consistent with studies done in Ethiopia [40] and Bangladesh [45]. As recommended by other researchers [38], community awareness interventions on the health facility delivery and birth preparedness promotion are required. The use of village health teams that has been indicated as a key intervention in translating health information to the rural communities should be strengthened in these communities [19, 46, 47].

Evidence from this study has indicated that the poor services at the health facilities, financial and transport problems, therefore, make women prefer giving birth at TBAs’ homes to health facilities since their (TBAs) services are nearer to them and the cost involved are not a burden to them. This linkage has also been highlighted in some of the studies done in LMICs [38, 45]. In fact, the role of the traditional birth attendants in helping mothers deliver in these rural communities was appreciated, a perception that has been reported in other studies [38, 39]. This, therefore, indicates the importance of addressing both facility and community challenges if the country is to improve skilled service utilization, which has been indicated as a cornerstone for reducing maternal and newborn death.

### Study strength and limitations

The strength of this study is that it uses baseline and follow-up data to assess the changes in the availability of services, supplies, and staff. In addition, the study combines both facility and community factors that influence the quality of care and service utilization, which is key in providing solutions aimed at addressing both demand and supply problems. The limitation of this study is that data were collected from rural health facilities and communities of Eastern Uganda. However, since most of the health facilities are in rural communities and often share same characteristics, these results will provide evidence on the state of maternal and newborn healthcare services in rural facilities.

## Conclusion

Although the user fee was abolished in all public health facilities in Uganda, the itching questions are: What is the quality of care at public health facilities? and how accessible are the healthcare services? The study has highlighted inadequacy in healthcare coverage resulting mainly from the poor quality of services and poor transport systems. Therefore, for universality of maternal and newborn services particularly in rural communities, there is a need to improve the quality of care and transport systems at community and health facility level. Drugs and equipment that are necessary at the time of birth should be sufficiently available. In addition, an interactive system that involves key stakeholders to monitor and provide appropriate measures such as resources and policies should be put in place.

## Abbreviations

ANC: Antenatal Care
APH: Antepartum haemorrhage
CEmOC: Comprehensive Emergency Obstetric Care
FGD: Focus Group Discussions
KIs: Key Informant Interviews
LMICs: Low and middle-income countries
MNH: Maternal and Newborn Health
PMTCT: Prevention of Mother To Child Transmission
PNC: Postnatal care
TBAs: Traditional Birth Attendants
WHO: World Health Organization.
